# Phantom-based acquisition time and image reconstruction parameter optimisation for oncologic FDG PET/CT examinations using a digital system

**DOI:** 10.1186/s12885-022-09993-4

**Published:** 2022-08-18

**Authors:** Pedro Fragoso Costa, Walter Jentzen, Alissa Brahmer, Ilektra-Antonia Mavroeidi, Fadi Zarrad, Lale Umutlu, Wolfgang P. Fendler, Christoph Rischpler, Ken Herrmann, Maurizio Conti, Robert Seifert, Miriam Sraieb, Manuel Weber, David Kersting

**Affiliations:** 1 Department of Nuclear Medicine, University Hospital Essen, West German Cancer Center (WTZ), University of Duisburg-Essen, Hufelandstrasse 55, 45147 Essen, Germany; 2 German Cancer Consortium (DKTK), Partner Site University Hospital Essen, Essen, Germany; 3 Department of Medical Oncology, University Hospital Essen, West German Cancer Center (WTZ), University Duisburg-Essen, 45147 Essen, Germany; 4 Department of Diagnostic and Interventional Radiology and Neuroradiology, University Hospital Essen, University Duisburg-Essen, 45147 Essen, Germany; 5 Siemens Medical Solutions USA, Inc., Knoxville, TN USA

**Keywords:** Positron emission tomography, FDG, Acquisition time, Silicon-based photomultiplier, Digital PET, Protocol optimisation, Lymphoma

## Abstract

**Background:**

New-generation silicon-photomultiplier (SiPM)-based PET/CT systems exhibit an improved lesion detectability and image quality due to a higher detector sensitivity. Consequently, the acquisition time can be reduced while maintaining diagnostic quality. The aim of this study was to determine the lowest ^18^F-FDG PET acquisition time without loss of diagnostic information and to optimise image reconstruction parameters (image reconstruction algorithm, number of iterations, voxel size, Gaussian filter) by phantom imaging. Moreover, patient data are evaluated to confirm the phantom results.

**Methods:**

Three phantoms were used: a soft-tissue tumour phantom, a bone-lung tumour phantom, and a resolution phantom. Phantom conditions (lesion sizes from 6.5 mm to 28.8 mm in diameter, lesion activity concentration of 15 kBq/mL, and signal-to-background ratio of 5:1) were derived from patient data. PET data were acquired on an SiPM-based Biograph Vision PET/CT system for 10 min in list-mode format and resampled into time frames from 30 to 300 s in 30-s increments to simulate different acquisition times. Different image reconstructions with varying iterations, voxel sizes, and Gaussian filters were probed. Contrast-to-noise-ratio (CNR), maximum, and peak signal were evaluated using the 10-min acquisition time image as reference. A threshold CNR value ≥ 5 and a maximum (peak) deviation of ± 20% were considered acceptable. 20 patient data sets were evaluated regarding lesion quantification as well as agreement and correlation between reduced and full acquisition time standard uptake values (assessed by Pearson correlation coefficient, intraclass correlation coefficient, Bland–Altman analyses, and Krippendorff’s alpha).

**Results:**

An acquisition time of 60 s per bed position yielded acceptable detectability and quantification results for clinically relevant phantom lesions ≥ 9.7 mm in diameter using OSEM-TOF or OSEM-TOF+PSF image reconstruction, a 4-mm Gaussian filter, and a 1.65 × 1.65 x 2.00-mm^3^ or 3.30 × 3.30 x 3.00-mm^3^ voxel size. Correlation and agreement of patient lesion quantification between full and reduced acquisition times were excellent.

**Conclusion:**

A threefold reduction in acquisition time is possible. Patients might benefit from more comfortable examinations or reduced radiation exposure, if instead of the acquisition time the applied activity is reduced.

**Supplementary Information:**

The online version contains supplementary material available at 10.1186/s12885-022-09993-4.

## Background

New-generation “digital” positron emission tomography/computed tomography (PET/CT) systems show a higher spatial and coincidence time resolution than previous-generation systems mainly because they use silicon-photomultipliers (SiPMs) that exhibit a higher detector sensitivity than previously used photomultiplier-tubes (PMTs) [1, 2]. Consequently, lesion detectability and image quality are improved [3–5]. Therefore, the acquisition time can be reduced while maintaining diagnostic image quality [6, 7].

Advantages of a reduced acquisition time include improved cost effectiveness and patient comfort, especially for pain-stricken, dyspnoeic, or paediatric patients [8, 9]. Moreover, motion artifacts can be reduced [10]. A typical clinical indication for ^18^F-FDG PET/CT scans is interim and final staging during chemotherapy of lymphoma patients [11]. As solid lymphomas belong to the most frequent malignancies in children, they are a common indication for paediatric PET scans [12, 13].

Alternatively, the amount of administered activity could be reduced, since, in a first approximation, a linear correlation between acquisition time and administered activity can be assumed [7, 14]. Thus, the radiation exposure for medical staff and patients could be reduced. Typical young lymphoma patients who respond to chemotherapy and show a high long-term survival could benefit from a reduced risk for secondary radiation-induced malignancy [15].

According to the current EANM guidelines for oncologic ^18^F-FDG PET/CT imaging [16] a typical acquisition time is 3 min per bed position, if activities of 2–3 MBq per kg patient weight are administered. We hypothesise that by application of a current-generation SiPM-based Biograph Vision PET/CT systems the administered activity can be reduced while maintaining lesion detectability and image quantification. A preliminary patient evaluation by our group indicated that a reduction by a factor of one-third is feasible while maintaining diagnostic quality [6]. However, comprehensive phantom measurements to confirm the hypothesis under defined conditions are, to the best of the authors’ knowledge, yet missing.

The aim of this study was to optimise the ^18^F-FDG PET acquisition time and image reconstruction parameters (image reconstruction algorithm, number of iterations, voxel size, Gaussian filter) derived from imaging of several phantoms, whose setup and preparation were based on clinical data of lymphoma patients. Moreover, previously acquired patient data are re-evaluated to validate the phantom results.

## Methods

### Phantom setup and preparation

To simulate nodal and extra-nodal lymphoma lesions, a soft-tissue tumour phantom and a bone-lung tumour phantom were used. Thorax and skeletal system are, among others, common localisations of extra-nodal lymphoma manifestations [17, 18]. Additionally, a line phantom was used for determination of spatial resolution.

To derive phantom conditions that mimic clinically realistic conditions, 16 randomly selected FDG PET/CT scans of clinical routine lymphoma patients were evaluated. For comparability, measured activity concentrations were normalised to patient weight and administered activity (assuming a 70-kg patient mass and an administered activity of 3 MBq/kg as recommended by current guidelines [16]); signal-to-background-ratios (SBRs) and volumes were estimated for a total of 31 lesions. Background activity concentrations were evaluated in horse-shoe-shaped volumes-of-interest (VOIs) surrounding the respective lesion; volumes were determined using a 3D-isocontour approach with a 50%-of-maximum threshold [16] or using CT data. 24/31 lesions were nodal lesions, the remaining 7/31 were bone lesions. Mean (minimum–maximum) activity concentration and volume were 12.2 (4.2–43.8) kBq/mL and 56.7 (0.7–328.1) mL, respectively. The mean (minimum–maximum) SBR was 4.0 (2.1–4.6). Based on these clinical data, a representative activity concentration of 15 kBq/mL and a SBR of 5:1 were selected. Since detectability and quantification is most challenging for small lesions, lesion volumes reflecting the lower range of volumes in the clinical evaluation were selected.

#### Soft-tissue tumour phantom

**Fig. 1:**
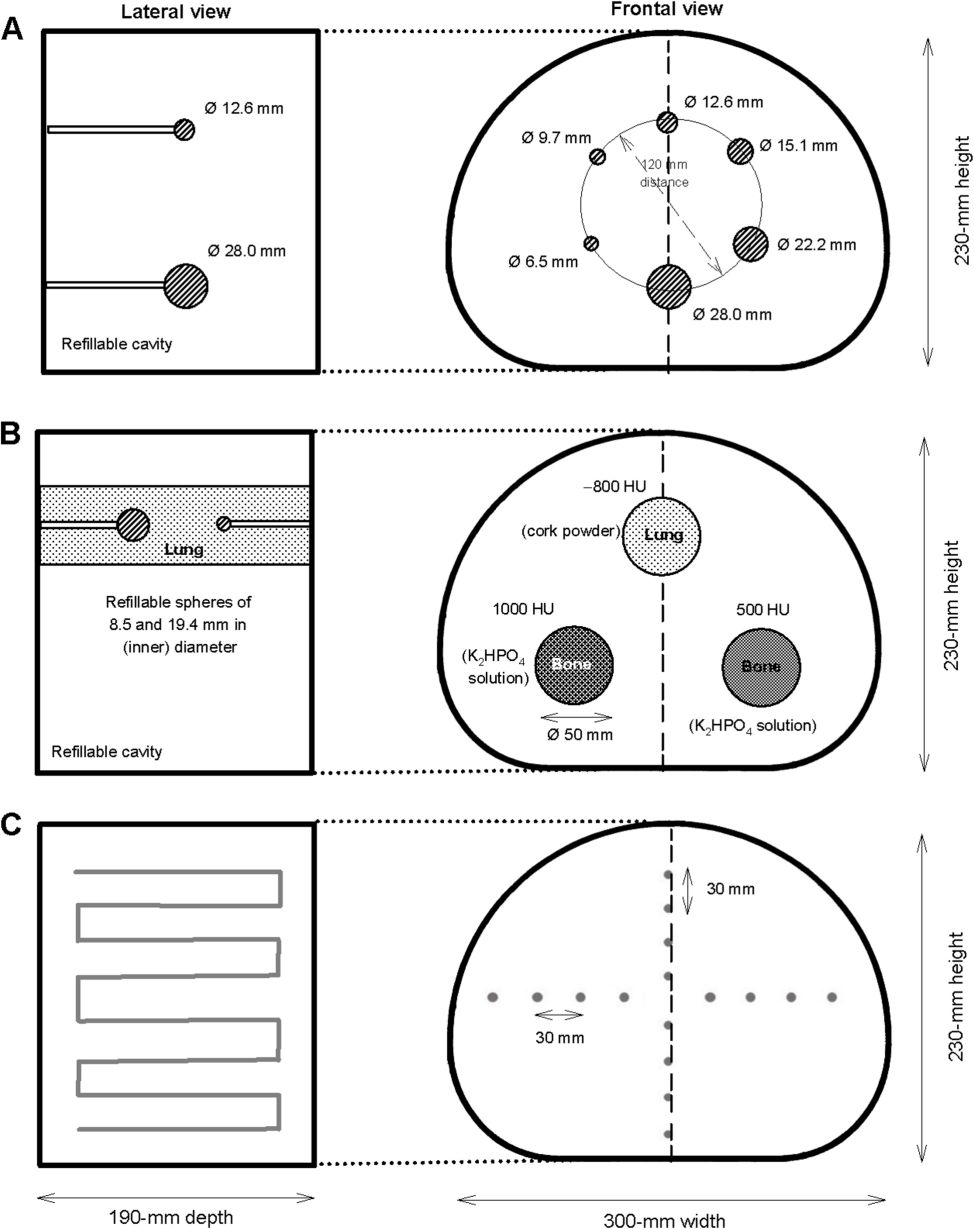
Frontal and lateral views (central plane) of a schematic representation of the soft-tissue (**A**), the bone-lung tumour phantom (**B**), and the resolution phantom (**C**)

The soft-tissue phantom consisting of a torso-shaped NEMA phantom (Data Spectrum Corporation, Durham, USA) that contains 6 small spheres (Fig. 1A) was designed to simulate hot lesions in a uniform warm background. Both spheres and phantom cavity were filled with radioactive solution*.* The largest sphere in the original NEMA phantom (37.0-mm diameter) was replaced by a small sphere (6.5-mm diameter). Thus, the resulting sphere diameters ranged from 6.5 mm to 28.0 mm (Fig. 1A). Each sphere was filled with ^18^F-FDG in aqueous solution. Of note, the second smallest sphere (9.7-mm diameter) represents a typical clinical threshold size for conspicuous lymph nodes [19].

#### Bone-lung tumour phantom

The bone-lung tumour phantom consisting of three cylindrical tubes inside the torso-shaped NEMA phantom (Fig. 1B) was designed to simulate hot pulmonary or osseous tumour lesions in a non-radioactive environment. To represent the CT density of lung-tissue (–800 HU), one tube was filled with cork powder (3 × 10^–4^ g/mm^3^-density, grain size 0.0–0.2 mm; Dumke Group, Rothenstein, Germany). To simulate the CT density of different kinds of bone lesions, two tubes were filled with dipotassium hydrogen phosphate (K_2_HPO_4_) solution in different concentrations. The calculated mass absorption coefficient for K_2_HPO_4_ at 500 keV is 0.0868 cm^2^/g [20] closely resembling the mass absorption coefficient for cortical bone of 0.0902 cm^2^/g [20]. Therefore, K_2_HPO_4_ is commonly used to simulate bone lesions in phantom investigations of attenuation correction in nuclear medicine imaging techniques [21, 22]. In the bone-lung tumour phantom, the resulting CT densities of 500 HU and 1000 HU reflect the spherical head of the humerus (538 HU) and the femoral shaft (1239 HU) [23]. Each tube contained two spheres (8.5-mm and 19.4-mm diameter) filled with ^18^F-FDG in aqueous solution. Spheres and phantom cavity were filled with radioactive solution. The tubes, however, contained no radioactive solution.

#### Resolution phantom

The resolution phantom contains multiple line sources consisting of polyethylene tubing (that are mounted into the abdominal torso phantom in orthogonal orientation to the transverse plane, Fig. 1C). The inner diameter of the line sources is 0.5 mm and they are looped back through the phantom to provide distances of 10 mm and 100 mm from the central axis of the field-of-view. They were filled with ^18^F-FDG in aqueous solution with an activity concentration of 7.1 MBq/mL; the total activity in the scanner was 21 MBq. For spatial resolution quantification with scatter, the phantom cavity was filled with non-radioactive water.

### PET data acquisition and image reconstruction

All phantom PET data were acquired on a SiPM-based Biograph Vision 600 PET/CT scanner (Siemens Healthineers, Erlangen, Germany) using a single bed position in list-mode. Detailed scanner specifications are given in Table 1. For attenuation correction, additional CT data were acquired (acquisition conditions: Care DOSE 4D, quality reference 160 mAs; CARE kV, quality reference 120 kV).

**Table 1:** Detailed specifications of the Biograph Vision PET/CT system. *LSO* Lutetium Oxyorthosilicate

	**Biograph Vision**
Detector material	LSO
Detector element dimension (mm^3)^	3.2 × 3.2 x 20
Detector elements per block	16 × 16
Total number of detector elements	60,800
Signal readout	SiPM (2 × 2 per block)
Axial field-of-view (mm)	263
Transaxial field-of-view (mm)	780
Plane spacing (mm)	1.65
Image planes	119
Coincidence time window (ns)	4.7
Energy window (keV)	435–585
Energy resolution (%)	9
System time resolution (ps)	210
NEMA sensitivity (kcps/MBq)	16.4

#### Soft-tissue and bone-lung tumour phantoms

PET data were acquired for 10 min in list-mode (one single bed position) and resampled into time frames from 30 to 300 s in 30-s increment to simulate different acquisition times. The 10-min acquisition time data were used as reference standard.

PET images were reconstructed using three-dimensional ordinary Poisson ordered-subset expectation maximization (OSEM) with or without time-of-flight option (TOF) and with or without point-spread-function modelling (PSF) resulting in four different combinations of reconstruction algorithms (i.e., OSEM, OSEM-TOF, OSEM-PSF, OSEM-TOF+PSF). Moreover, different numbers of iterations (4, 6, and 8 for TOF-based and 10,12, and 14 for non-TOF reconstruction algorithms) were evaluated. For both non-TOF and TOF-based reconstruction groups, the smallest investigated number of iterations reflects the manufacturer’s recommendation for the respective reconstruction algorithm. Of note, the number of subsets was 5 and was fixed in all image reconstructions.

The applied matrices (220 or 440) and Gaussian filters (2 mm or 4 mm) resulted in 4 different combinations of voxel sizes and Gaussian filters (i.e., 1.65 × 1.65 x 2.00-mm^3^ voxel size and 2-mm Gaussian filter, 3.30 × 3.30 x 3.00-mm^3^ voxel size and 2-mm Gaussian filter, 1.65 × 1.65 x 2.00-mm^3^ voxel size and 4-mm Gaussian filter, 3.30 × 3.30 x 3.00-mm^3^ voxel size and 4-mm Gaussian filter).

#### Resolution phantom

To determine the spatial resolution at high counting statistics, the acquisition time was increased to 30 min. PET data were reconstructed using OSEM-TOF with for 4 iterations and OSEM-TOF+PSF with 4 iterations only. Two different spatial resolutions were defined. For estimation of the *system spatial resolution,* an 880 matrix was applied (resulting voxel size of 0.83 × 0.83 x 2.00 mm^3^) without Gaussian filter. The *clinical spatial resolution* further includes Gaussian filtering (2-mm or 4-mm filter level) and was calculated from the *system spatial resolution* as the convolution of two Gaussian functions [24] (see Eq. 4).

### Analyses of phantom images

#### Soft-tissue and bone-lung tumour phantom

Three activity concentration values were determined for the spherical objects and one activity concentration for the background. Sphere average activity concentrations were determined in spherical VOIs whose diameter matched that of the real spheres. Sphere average activity concentrations were applied (see below) to calculate the contrast-to-noise ratio (CNR). Maximum activity concentrations were derived within the sphere boundary; peak activity concentrations were ascertained in peak VOIs (sphere of 1 mL-volume) that were placed at the centre of the respective spherical phantom insert. Peak and maximum activity concentrations were used to assess the activity concentration accuracy at different acquisition times (see below). For the soft-tissue tumour phantom only, image noise was evaluated. For this purpose, the average activity concentration and its standard deviation were determined in a background VOI. The background VOI consisted of 60 two-dimensional 37-mm diameter circular background regions-of-interest that were placed in the central plane of the spherical phantom inserts and the two adjacent planes in each direction (12 regions-of-interest in each plane) according to NEMA "Standard for Performance Measurements of Positron Emission Tomographs NU 2–2012″ [25].

To evaluate the lesion detectability, the contrast-to-noise ratio (CNR) was calculated for the spherical inserts in the soft-tissue tumour phantom as previously described [26]:

1 $$\begin{array}{c}CNR = \frac{{C}_{\mathrm{avg}}\ - \ {C}_{\mathrm{bgr}}}{{SD}_{\mathrm{bgr}}}\end{array}$$

using the average sphere activity concentration (denoted by *C*_avg_), the average activity concentration in the background VOI (denoted by *C*_bgr_), and the standard deviation of the activity concentration in the background VOI (denoted by *SD*_bgr_).

For both the soft-tissue tumour and the bone-lung tumour phantom, the accuracy of activity concentration measurements in short-acquisition time images was evaluated. For this purpose, maximum and peak activity concentration ratios ACR_max_ and ACR_peak_ were calculated using the 10-min scan as reference:

2 $$\begin{array}{c}{\mathrm{ACR}}_{\mathrm{max}} = \frac{{C}_{\mathrm{max}}}{{C}_{\mathrm{max},\mathrm{ref}}}\end{array}$$

and

3 $$\begin{array}{c}{\mathrm{ACR}}_{\mathrm{peak}} = \frac{{C}_{\mathrm{peak}}}{{C}_{\mathrm{peak},\mathrm{ref}}}\end{array}$$

defining the maximum (peak) sphere activity concentration, *C*_max_ (*C*_peak_), at varying acquisition times and the respective maximum (peak) activity concentration in the 10-min reference image *C*_max,ref_ (*C*_peak,ref_).

Of note, maximum and peak activity concentrations can be regarded as surrogates of the clinically established maximum and peak standardised uptake values SUV_max_ and SUV_peak_. SUV_max_ and SUV_peak_ are used for patient PET data to describe activity concentration measurements, which are normalised to patient mass and administered activity. Therefore, they cannot be defined for phantom evaluations.

CNR and activity concentration ratios were evaluated as a function of the acquisition time. According to the Rose criterion, spheres with CNR ≥ 5 were considered as visible. The Rose criterion was originally derived from quantum effects in the visual process [27] and is commonly applied to define visible objects in PET imaging [26, 28]. An activity concentration percentage deviation range of ± 20% was considered acceptable based on published data on the test–retest reliability of ^18^F-FDG PET data [29, 30].

#### Resolution phantom

The system spatial resolution was determined as previously described [24] for the purpose of evaluating the image reconstruction parameters. In brief, the spatial resolution was estimated at three transversal positions (at the centre and at one-fourth of the PET scanner’s field-of-view in each direction). Gaussian functions were fitted to radial and tangential activity concentration profiles (that were determined at 10-mm and 100-mm distance from the centre at each transversal position) to estimate the respective full width at half maximum (FWHM). The system spatial resolution (FWHM_sys_) was defined as mean of all estimated FWHM values.

The clinical spatial resolution FWHM_clin_ was determined from the estimated system resolution FWHM_sys_ and the respective Gaussian filter size (FWHM_Gauss_). The effective FWHM of the convolution of the two Gaussian functions can be calculated using the following equation [24]:

4 $$\begin{array}{c}{\mathrm{FWHM}}_{\mathrm{clin}} = \sqrt{{\mathrm{FWHM}}_{\mathrm{sys}}^{2} + {\mathrm{FWHM}}_{\mathrm{Gauss}}^{2}}\end{array}$$

Spatial resolution determination was performed for a voxel size of 0.83 × 0.83 x 2.00 mm^3^ to achieve the highest possible resolution.

### Patient data evaluation

To validate the phantom results, previously published patient data [6] were re-evaluated. In brief, ^18^F-FDG PET data of 20 lymphoma patients were acquired on a Biograph Vision 600 PET/CT system separately at a clinical standard and at an approximately three-fold reduced total PET acquisition time. Image reconstruction was performed using OSEM-TOF and OSEM-TOF+PSF, respectively, a voxel size of 3.30 × 3.30 x 3.00 mm^3^ and a 4-mm Gaussian filter. For a total of 30 lesions, SUV_max_ and SUV_peak_ values were determined at standard and reduced acquisition times. These data were subjected to extended statistical analyses. Moreover, maximum intensity projection (MIP) PET images of a single patient example are presented.

### Software/statistics

PET image analysis and VOI segmentation was performed using PMOD 4.202 (PMOD Technologies, Zurich, Switzerland). All statistical computations were performed using R 4.0.3 (R Foundation for Statistical Computing, Vienna, Austria, www.R-project.org). To describe the correlation between short and full acquisition time SUVs in a linear regression model, the Pearson correlation coefficient (PCC) was calculated. The two-way mixed effect model intraclass correlation coefficient (ICC) was used to describe the intra-test correlation [31, 32]; lower and upper confidence bounds were determined as defined by Shrout and Fleiss [31]. Following Koo et al. (28), an ICC ≥ 0.90 indicates excellent correlation. Bland–Altman analyses [33] and Krippendorff’s alpha [34] were used to evaluate the intra-test agreement of SUVs.

## Results

### Soft-tissue tumour phantom

**Fig. 2:**
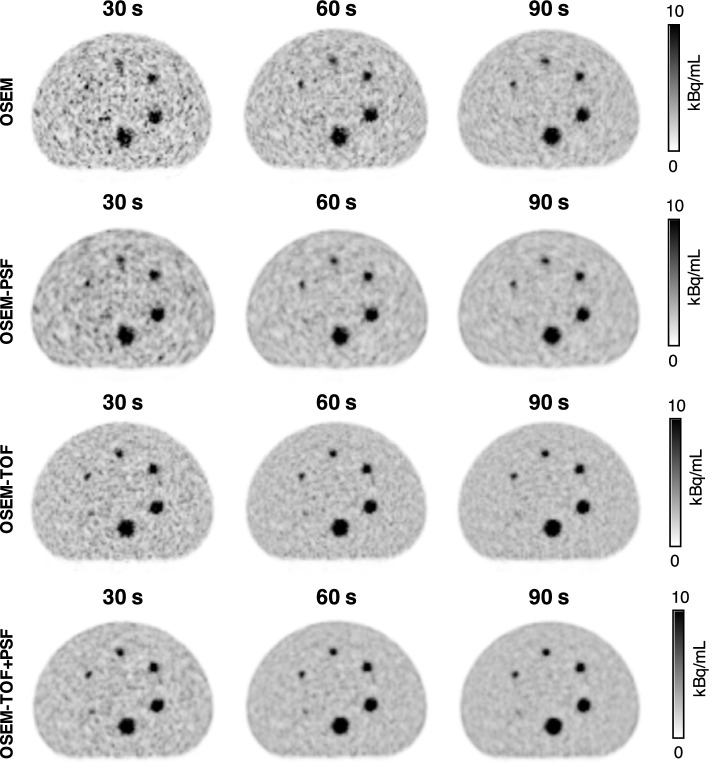
Exemplary collection of PET images of the soft-tissue tumour phantom for different acquisition times and reconstruction algorithms. All images were reconstructed with a voxel size of 3.30 × 3.30 x 3.00 mm^3^, a 2-mm Gaussian filter, and the smallest investigated number of iterations (10i for non-TOF and 4i for TOF-based image reconstructions). The diameters of the spherical phantom inserts were 6.5 mm, 9.7 mm, 12.6 mm, 15.1 mm, 22.2 mm, and 28.0 mm (clockwise starting from bottom-left)

A visual examination of the images revealed a good agreement with the CNR threshold value of CNR ≥ 5. Figure 2 exemplarily shows PET images of the soft-tissue tumour phantom for different acquisition times and image reconstruction algorithms.

#### Contrast-to-noise ratio

**Fig. 3:**
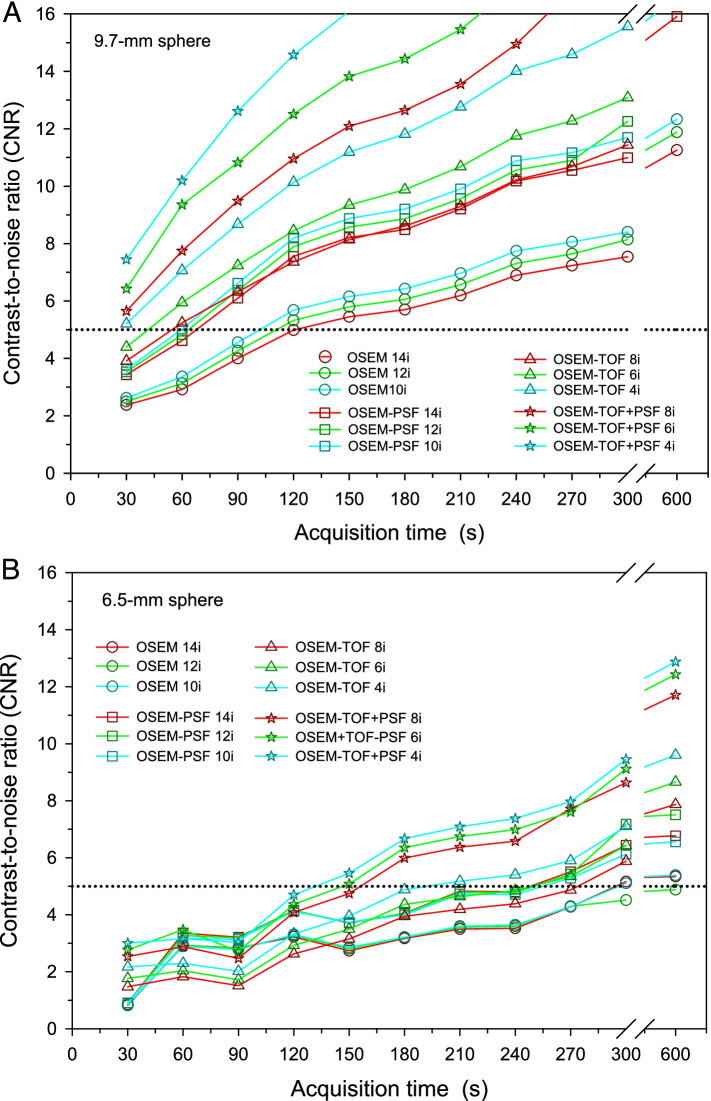
CNR of the 9.7-mm (**A**) and 6.5-mm (**B**) diameter soft-tissue tumour phantom spheres as a function of the acquisition time for all investigated image reconstruction algorithms and numbers of iterations (3.30 × 3.30 x 3.00-mm^3^ voxel size and 2-mm Gaussian filter). The sphere activity concentration was 15 kBq/mL and the SBR was 5:1 (these parameters were selected to represent clinical data). The dashed horizontal line indicates a CNR threshold value of 5

For sphere diameters > 10 mm, the CNR was ≥ 5 for all investigated acquisition times, reconstruction algorithms and number of iterations. For the 6.5-mm and the 9.7-mm sphere, the detectability was dependant on the acquisition conditions (Fig. 3, CNR curves not shown for sphere diameters > 10 mm). The smallest lesion in the evaluation of patient images that was performed to select the phantom conditions was 0.7 ml. Assuming a spherical lesion, this results in a corresponding sphere diameter of 11 mm. Hence, the following evaluation is mainly performed for the 9.7-mm sphere that best matches the minimal size of patient lesions.

For both the 6.5- and the 9.7-mm sphere and for all acquisition conditions, the highest CNR values were observed for the lowest numbers of iterations, i.e., *n* = 10 for non-TOF and *n* = 4 for TOF-based reconstruction algorithms. OSEM-TOF+PSF performed best, followed by OSEM-TOF, OSEM-PSF, and OSEM in descending order applying a standard voxel size of 3.30 × 3.30 x 3.00 mm^3^ and Gaussian filter of 2 mm (Fig. 3). Therefore, the detailed evaluation was only performed for TOF-based reconstructions (with lowest number of iterations of 4): For the 9.7-mm sphere, the CNR was ≥ 5 for all evaluated acquisition times (Fig. 3A). Of note, the smallest (6.5-mm) sphere was only detectable at larger acquisition times of 210 s (OSEM-TOF, 4 iterations) and 150 s (OSEM-TOF+PSF, 4 iterations), respectively (Fig. 3B).

**Fig. 4:**
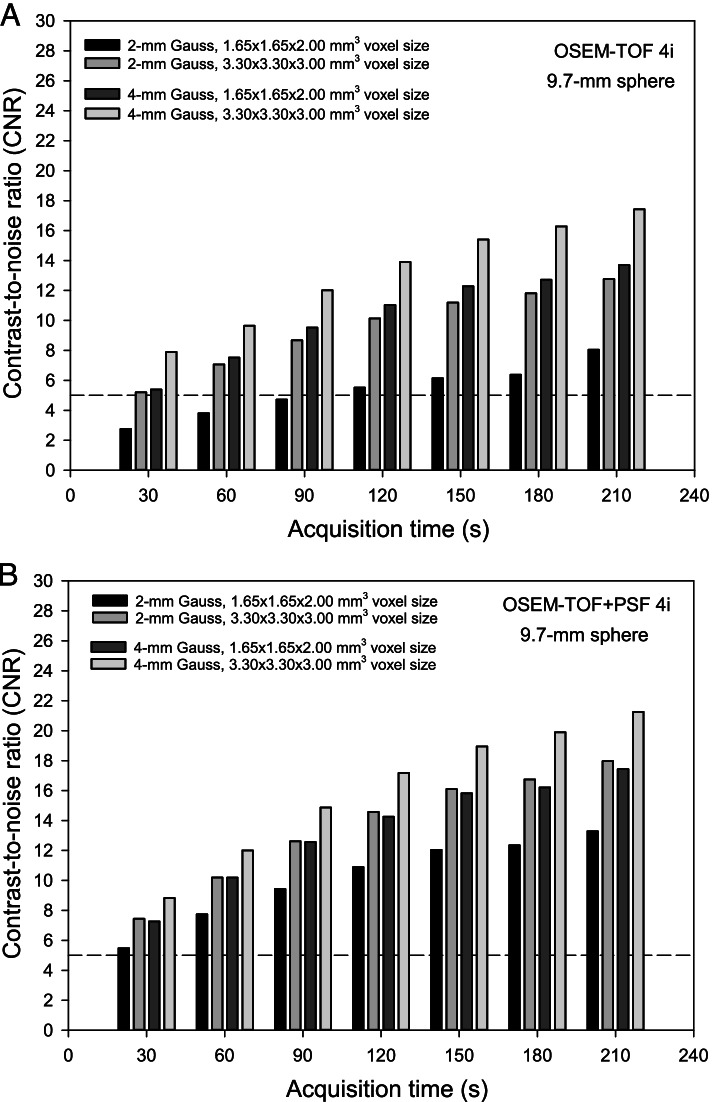
CNR of the 9.7-mm diameter soft-tissue tumour phantom sphere as a function of the acquisition time for the different investigated combinations of voxel sizes and Gaussian filters, separately for OSEM-TOF (**A**) and OSEM-TOF+PSF (**B**) image reconstructions. The dashed horizontal line indicates a CNR threshold value of 5

Varying voxel size and Gaussian filter, best results were achieved for a voxel size of 3.30 × 3.30 x 3.00 mm^3^ and a 4-mm Gaussian filter (Fig. 4). For a 30-s to 90-s acquisition time, the detectability criterion was met for all voxel sizes and Gaussian filters except for OSEM-TOF using a small voxel size of 1.65 × 1.65 x 2.00-mm^3^ and a low Gaussian smoothing level of 2 mm. For larger acquisition times, the CNR was ≥ 5 for all acquisition conditions.

#### Quantification

**Fig. 5:**
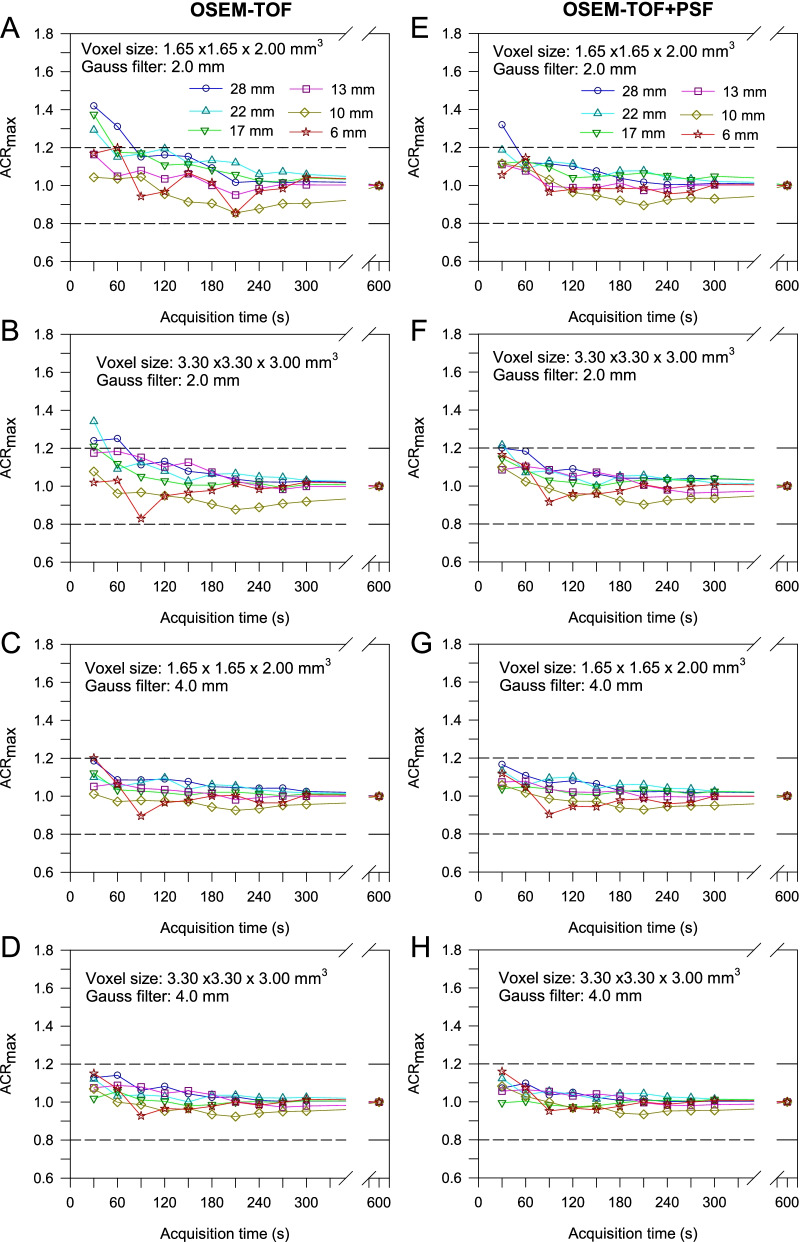
Maximum activity concentration ratio for the small-tumour phantom as a function of the acquisition time by reference to the 10-min acquisition time PET images separately for the different investigated combinations of voxel sizes, Gaussian filters, and OSEM-TOF (**A**-**D**) or OSEM-TOF+PSF (**E**–**H**) image reconstructions. Data for all investigated spheres are presented. Dashed horizontal lines indicate the ± 20% deviation (acceptance criterion)

For the soft-tissue tumour phantom, best maximum activity concentration quantification results were achieved for a voxel size of 3.30 × 3.30 x 3.00 mm^3^ and a 4-mm Gaussian filter, followed by a voxel size of 1.65 × 1.65 x 2.00 mm^3^ and a 4-mm Gaussian filter (Fig. 5C and D for OSEM-TOF and Fig. 5G and H for OSEM-TOF+PSF). For both OSEM-TOF+PSF and OSEM-TOF (3.30 × 3.30 x 3.00-mm^3^ or 1.65 × 1.65 x 2.00-mm^3^ voxel size and 4-mm Gaussian filter) the maximum activity concentration quantification acceptance criterion was met for a 30-s acquisition time. For a 2-mm Gaussian filter (Fig. 5A and B for OSEM-TOF and Fig. 5E and F for OSEM-TOF+PSF), acquisition times of 60 to 120 s were necessary to fulfil the acceptance criterion depending on voxel size and reconstruction algorithm. The evaluation of the peak activity concentration quantification accuracy showed an acceptable quantification accuracy for a 30-s acquisition time for all examined reconstruction parameters (Supplemental Figure S1).

### Bone-lung Tumour Phantom

**Fig. 6:**
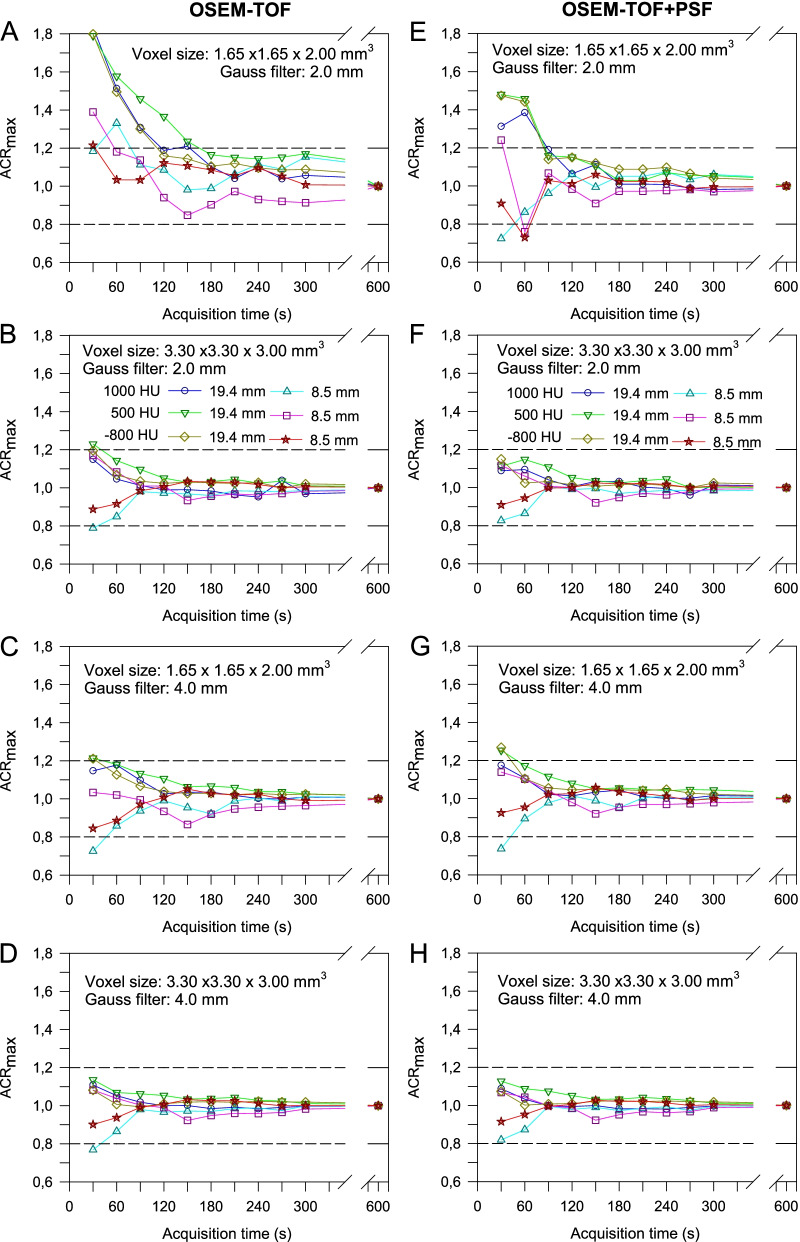
Maximum activity concentration ratio for the bone-lung phantom as a function of the acquisition time by reference to the 10-min acquisition time PET images separately for the different investigated combinations of voxel sizes, Gaussian filters, and OSEM-TOF (**A**-**D**) or OSEM-TOF+PSF (**E**–**H**) image reconstructions. Data for all investigated spheres/density regions are presented. Dashed horizontal lines indicate the ± 20% deviation (acceptance criterion)

For the bone-lung tumour phantom, best results were achieved for a voxel size of 3.30 × 3.30 x 3.00 mm^3^ and a 4-mm Gaussian filter (Fig. 6D and H), followed by a voxel size of 1.65 × 1.65 x 2.00 mm^3^ and a 4-mm Gaussian filter (Fig. 6C and G). For OSEM-TOF+PSF (3.30 × 3.30 x 3.00-mm^3^ voxel size and 4-mm Gaussian filter) the maximum activity concentration quantification acceptance criterion was met for a 30-s acquisition time, whereas for OSEM-TOF a 60-s acquisition time was required for accurate quantification in all three density regions. For a 2-mm Gaussian filter and a voxel size of 1.65 × 1.65 x 2.00 mm^3^ (Fig. 6A and E), acquisition times of 90 s (OSEM-TOF+PSF) and 150 s (OSEM-TOF) were necessary to fulfil the acceptance criterion. When increasing the voxel size to 3.30 × 3.30 x 3.00 mm^3^ while maintaining the 2-mm Gaussian filter (Fig. 6B and F), acquisition times of 30 s (OSEM-TOF+PSF) and 60 s (OSEM-TOF) were necessary. Similar results were achieved for the evaluation of the peak activity concentration quantification accuracy (Supplemental Figure S2). Of note, to meet the peak activity concentration acceptance criterion, even for the best performing reconstruction parameters (OSEM-TOF+PSF, 3.30 × 3.30 x 3.00 mm^3^-voxel size and 4-mm Gaussian filter) a 60-s acquisition time was necessary.

### Resolution phantom

System spatial resolution (average FWHM_sys_) was 3.6 ± 0.3 mm for OSEM-TOF and 2.9 ± 0.2 mm for OSEM-TOF+PSF. Clinical spatial resolutions (FWHM_clin_) were 4.0 mm (OSEM-TOF) and 3.5 mm (OSEM-TOF+PSF) for a 2-mm Gaussian filter and 5.4 mm (OSEM-TOF) and 4.9 mm (OSEM-TOF+PSF) for a 4-mm Gaussian filter. All resolution phantom results are listed in Table 2.

**Table 2:** Detailed system spatial resolution results for the resolution phantom. All spatial resolutions were derived for a voxel size of 0.83 × 0.83 x 2.00 mm^3^

	Gaussian filter	OSEM-TOF	OSEM-TOF + PSF
FWHM_sys_	-	3.6 mm	2.9 mm
Standard deviation of FWHM_sys_	-	0.3 mm	0.2 mm
FWHM_clin_	2 mm	4.0 mm	3.5 mm
FWHM_clin_	4 mm	5.4 mm	4.9 mm

### Evaluation of patient data

**Fig. 7:**
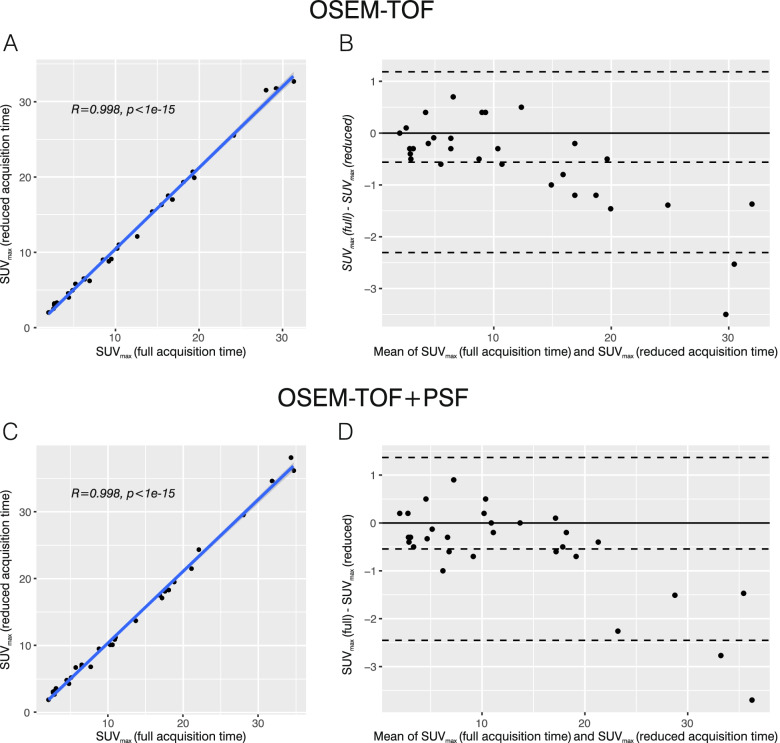
Analysis of correlation and agreement of patient data (*n* = 31 lesions). Scatter plot (**A**) and Bland–Altman analysis (**B**) for SUV_max_ of reduced acquisition time versus SUV_max_ of full acquisition time PET images for OSEM-TOF reconstructions. Scatter plot (**C**) and Bland–Altman analysis (**D**) for SUV_max_ of reduced acquisition time versus SUV_max_ of full acquisition time PET images for OSEM-TOF+PSF image reconstructions

**Fig. 8:**
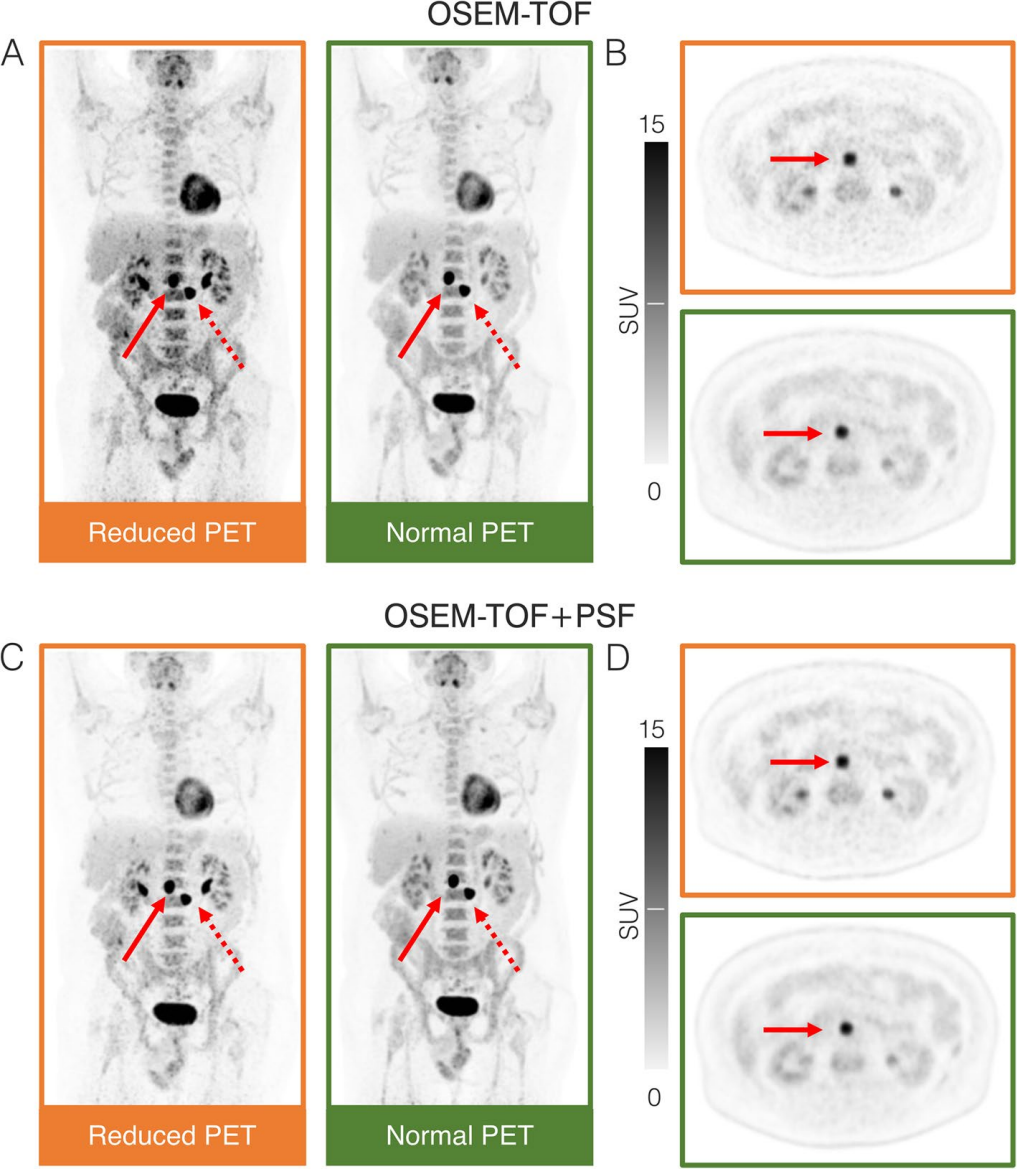
MIP (**A**&**C**) and transversal slice (**B**&**D**) PET images of a Non-Hodgkin Lymphoma patient who underwent FDG PET/CT for re-staging after chemotherapy. Two retroperitoneal lymphoma manifestations were detected in both reduced (orange) and full (green) acquisition time PET images. For the cranial lesion (solid arrows), SUV_max_ was 31.7 (34.6) for the reduced and 29.2 (31.8) for the full acquisition time images using OSEM-TOF (OSEM-TOF+PSF) image reconstruction. For the caudal lesion (dashed arrows), SUV_max_ was 32.7 (36.2) for the reduced and 31.3 (34.7) for the full acquisition time images using OSEM-TOF (OSEM-TOF+PSF) image reconstruction. Of note, differences in kidney activity distribution (urine activity) are caused by slightly different time-points of PET imaging

To evaluate the phantom results in a clinical context, data from a previously published patient study [6] were re-evaluated. Quantification results were compared between clinical standard and approximately three-fold reduced acquisition time PET images of lymphoma patients. For an inter-patient comparability, clinically established SUV_max_ and SUV_peak_ were compared instead of absolute activity concentration values. Overall, agreement and correlation between short and full acquisition time SUV_max_ values were excellent for both OSEM-TOF and OSEM-TOF+PSF reconstructed images (Fig. 7). Detailed agreement and correlation analyses results (indicating PCC, ICC, Krippendorff’s alpha and Bland–Altman bias) are presented in Table 3. Likewise, agreement and correlation of SUV_peak_s were excellent for both OSEM-TOF and OSEM-TOF+PSF reconstructed images (Supplemental Figure S3 and Table 3). To indicate visual differences between short and full acquisition time PET images, a patient example is presented in Fig. 8.

**Table 3:** Detailed agreement and correlation analysis results for evaluated patient lesions. Standard and reduced acquisition time SUV_max_ and SUV_peak_  are compared

	OSEM-TOF	OSEM-TOF + PSF
PCC (95%-CI) for SUV_max_	0.998 (0.995 to 1.000)	0.998 (0.995 to 1.00)
ICC (lower bound – upper bound) for SUV_max_	0.994 (0.978 to 0.997)	0.994 (0.983 to 0.998)
Krippendorff’s alpha for SUV_max_	0.994	0.993
Bland–Altman bias (95%-CI) for reduced acquisition time SUV_max_	–0.56 (–0.90 to -0.22)	–0.54 (–0.91 to –0.17)
PCC (95%-CI) for SUV_peak_	0.998 (0.995 to 0.999)	0.998 (0.994 to 0.999)
ICC (lower bound – upper bound) for SUV_peak_	0.993 (0.979 to 0.997)	0.993 (0.980 to 0.997)
Krippendorff’s alpha for SUV_peak_	0.995	0.996
Bland–Altman bias (95%-CI) for reduced acquisition time SUV_peak_	–0.39 (–0.65 to -0.14)	–0.41 (–0.70 to -0.12)

## Discussion

The phantom study results indicate that the acquisition time could be reduced to 60 s per bed position (or 2.2 mm/s in continuous-bed-motion mode [35]) for ^18^F-FDG-PET/CT scans on the SiPM-based Biograph Vision while maintaining lesion detectability and accurate image quantification within acceptable limits. This is an approximately threefold reduction in acquisition time compared to commonly applied and recommended PET acquisition protocols [7, 16].

Several clinical evaluations for ^18^F-FDG and ^68^ Ga-PSMA suggest that an acquisition time reduction by a factor of one-third is feasible using an SiPM-based PET/CT system [6, 7]. In an evaluation by Alberts et al. [34], all lesions were detected at 50%-reduced acquisition time images, whereas 2/100 lesion were missed in 75%-reduced acquisition time images. However, a literature review revealed no published phantom study under clinically derived conditions to analyse the minimum possible acquisition time for oncologic ^18^F-FDG PET imaging.

Previously published phantom studies investigating SiPM-based PET/CT systems mostly use NEMA phantoms for system characterisation [2, 36–40] and do not investigate effects of a reduced acquisition time. One study by Gnesin et al. [36] reports a 40% to 70%-reduced time-activity-product for SiPM based PET systems indicating the possibility to reduce administered activity or acquisition time. A more sophisticated phantom study was performed by Surti et al. [41], who use a torso phantom and a cylindrical phantom to simulate lung and liver regions; lesions were scanned in air and later virtually embedded into the phantom data. They used the area under the localized receiver-operating-characteristic curve as metric to show that, compared to the PMT-based Biograph mCT, the imaging time could be reduced by a factor of 4–6 on the Vision [41]. However, no analysis of different reconstruction algorithms, number of iterations, voxel sizes, and Gaussian filters was performed in that study [41].

In this study, we used different phantoms and acquisition conditions derived from clinical lymphoma patient data to investigate a reduced acquisition time and select most beneficial reconstruction parameters. First, the CNR was investigated among different reconstruction algorithms and numbers of iterations at a standard voxel size of 3.30 × 3.30 x 3.00 mm^3^ and standard Gaussian smoothing level of 2 mm (Fig. 3). These evaluations were performed for the soft-tissue tumour phantom resembling nodal lymphoma lesions. TOF-based outperformed non-TOF image reconstruction algorithms and were selected for further investigation. TOF-based image reconstruction benefits in particular from the high coincidence timing resolution of SiPM-based PET systems [42]. Best results were achieved for the lowest number of iterations of 4 (in agreement with the manufacturer’s recommendation). Additional iterations led to reduced CNR values indicating increased noise; thus, convergence at 4 iterations is suggested [43]. Fast convergence of the iteration process was previously described for TOF-based image reconstruction on SiPM-based systems [44]. Of note, the further evaluations were based on the 9.7-mm diameter sphere that mimics the lower size limit of lesions in the previously performed patient evaluation and, therefore, represents the most challenging clinically realistic conditions. Likewise, the size of nodal lymphoma lesions was described as typically ≥ 10 mm [45].

Next, voxel size and level of the applied Gaussian filter were varied. Regarding detectability, small filter levels result in higher spatial resolution facilitating the detection of small lesions [46]. Regarding lesion quantification, Gaussian filtering can be used, for instance, to suppress Gibbs edge artefacts [47]. Best CNR values (for the representative 9.7-mm diameter sphere) were achieved for a 3.30 × 3.30 x 3.00-mm^3^ voxel size and a 4-mm Gaussian filter, followed by a 1.65 × 1.65 x 2.00-mm^3^ voxel size and a 4-mm Gaussian filter (Fig. 4) indicating an adequative detectability for a lesion size at the lower size range of typical clinical lesions. Of note, the Rose criterion (CNR ≥ 5) was met for all combinations and acquisition times except for 30-s to 90-s acquisition times at a 1.65 × 1.65 x 2.00-mm^3^ voxel size and a 2-mm Gaussian filter, and non-PSF image reconstruction.

An investigation of the quantification performance revealed that a 2-mm Gaussian filter smoothing resulted in inadequate quantification, particularly for larger lesions, at short acquisition times (Fig. 5). TOF-based image reconstruction and PSF-modelling can overcompensate for very low count statistics and lead to an overestimation of activity concentration values [48, 49]. To simulate typical extra-nodal lymphoma lesions, the evaluation was extended to the bone-lung tumour phantom and comparable results were observed (Fig. 6). Differences in CT densities compared to soft tissue can influence the scatter contribution. This might affect the PET scanner’s scatter correction that is based on tail-fitting to the PET data sinograms and, consequently, the lesion quantification performance.

Applying a Gaussian filter of 4 mm, at 60-s acquisition time satisfactory results were achieved for all phantom inserts at both 1.65 × 1.65 x 2.00-mm^3^ and 3.30 × 3.30 x 3.00-mm^3^ voxel sizes with favourable quantification performance for the larger voxel size. At 30-s acquisition time, quantification of maximum and/or peak activity concentration of the small lung-phantom insert in 1000-HU background was unsatisfactory, even for the best performing reconstruction parameters. Increasing the filter level from 2 to 4 mm resulted in a degradation of spatial resolution for OSEM-TOF (OSEM-TOF+PSF) from 4.0 mm (3.5 mm) to 5.4 mm (4.9 mm). Following the dependency of the system resolution on the sampling frequency (Huang criterion) [50], the spatial resolution could have been improved by decreasing the voxel size to 1.65 × 1.65 x 2.00-mm^3^. However, for the smaller voxel sizes the quantification accuracy was compromised, particularly for smaller acquisition times (Figs. 5 and 6). Moreover, the detectability of the 9.7-mm sphere was limited (CNR<5) for a 1.65 × 1.65 x 2.00-mm^3^ voxel size, a 2-mm Gaussian filter, and non-PSF image reconstruction as described above.

An acquisition time of 60 s per bed position signifies a threefold decrease to a clinically established acquisition time of 180 s per bed position [7]. A re-evaluation of previously acquired lymphoma patient PET data (acquired on the Biograph Vision at standard and 2.75-fold reduced acquisition times) was performed to infer on the applicability of the phantom results in a clinical setting. A detailed statistical evaluation of lesion quantification revealed an excellent correlation and agreement for SUV_max_ and SUV_peak_ between full and reduced acquisition time images for both OSEM-TOF and OSEM-TOF+PSF reconstructed images (Fig. 7, Supplemental Figure S3, and Table 3). Outliers in the Bland–Altman plots were observed for large SUVs (Fig. 7 and Supplemental Figure S3). They arise, as in the analysis a large range of SUVs (more than one magnitude) was covered. Thus, for larger SUVs, the absolute difference in SUV can be larger than for lower SUVs, even if the relative difference is low and they present as outliers in the Bland–Altman plot. The maximum percentage deviations were 18.5% (SUV_max_ OSEM-TOF), 17.5% (SUV_max_ OSEM-TOF+PSF), 14.1% (SUV_peak_ OSEM-TOF), and 13.7% (SUV_max_ OSEM-TOF+PSF), respectively. Hence, all deviations were below the 20% threshold for repeatability of ^18^F-FDG PET SUV measurements that was derived in an metanalysis of test–retest variability studies [29].

In summary, our data indicate that using an SiPM-based Biograph Vision PET/CT system an acquisition time of 60 s per bed position is sufficient to fulfill the lesion detectability and quantification acceptance criteria, if OSEM-TOF or OSEM-TOF+PSF image reconstruction, a 4-mm Gaussian filter, and a 1.65 × 1.65 x 2.00-mm^3^ or 3.30 × 3.30 x 3.00-mm^3^ voxel size are applied. For a further reduction in acquisition time to 30 s, not all acceptance criteria for all phantoms are met. This is in line with a published clinical evaluation of 100 lesions, in which all lesions were detected at 60 s acquisition time, whereas at 30 s acquisition time single lesions were missed [34]. Moreover, comparable results indicating a threshold of 60 s per bed position were also obtained in phantom and clinical studies investigating ^68^Ga-PSMA-11 PET/CT for imaging of prostate cancer patients [51, 52].

Different hardware and software improvements may lead to a further decrease in examination time/administered activity for whole-body PET examinations in the future. For example, total-body PET systems can enable reduced acquisition times [53–55]. In addition, artificial intelligence methods can be applied for PET image reconstruction and post-reconstruction. Several approaches demonstrated the capability of deep-learning neural networks to enhance low-count PET images [10, 56–58]. One software solution was already approved by the FDA and is commercially available (SubtlePET, Subtle Medical, Menlo Park, CA).

As typical PET acquisition times are long, for example, in comparison to CT acquisitions, PET imaging is influenced by patient motion including respiratory motion or patient movement. The visual impact of motion artifacts can be increased for high-resolution PET (as offered by SiPM-based PET) and for small lesions [59]. Moreover, attenuation correction can be deteriorated due to misalignment between PET and CT images. To reduce motion artifacts arising from patient movements, shorter PET acquisition times as enabled by SiPM-based systems can be of particular benefit. Influence of patient motion was not investigated in this phantom study. In the patient evaluation, the correlation between quantification results in reduced- and full-acquisition time PET images was excellent (Fig. 7, Supplemental Figure S3 and Table 3) indicating a low influence of motion artifacts in the investigated set of typical clinical lesions. In future, the implementation of total-body PET systems can be of further benefit regarding patient motion as, like in CT imaging, PET images could be acquired in respiratory breaks. Moreover, motion-correction can be performed by software- or hardware-driven approaches [60, 61].

The study faces several limitations. First, the phantoms are anthropomorphic to a limited extent. In contrast to the homogenous phantom conditions, real lymphoma manifestations can be non-spherical, inhomogeneous and of variable signal-to-background ratio. Second, the bone-lung tumour phantom represents homogeneous non-radioactive background which also deviates from real conditions. Third, the evaluation of patient data was only performed for a single choice of Gaussian filter and voxel size.

## Conclusions

Based on the phantom results, an acquisition time of 60 s per bed position yields acceptable detectability and quantification results for OSEM-TOF or OSEM-TOF+PSF image reconstruction using a 4-mm Gaussian filter and a 1.65 × 1.65 x 2.00-mm^3^ or 3.30 × 3.30 x 3.00-mm^3^ voxel size on an SiPM-based PET/CT system. The corresponding threefold reduction in acquisition time coincides with previously published clinical data for FDG and PSMA PET scans. Patients might benefit from more comfortable examinations or reduced radiation exposure, if instead of reduced acquisitions times the applied activity is reduced. Larger clinical studies are warranted for further evaluation.

## Supplementary Information

**Additional file 1: Supplemental Figure S1.** Peak activity concentration ratio for the small-tumour phantom as a function of the acquisition time by reference to the 10-min acquisition time PET images separately for the different investigated combinations of voxel sizes, Gaussian filters, and OSEM-TOF (A-D) or OSEM-TOF+PSF (E-H) image reconstructions. Data for all investigated spheres are presented. Dashed horizontal lines indicate the ±20% deviation (acceptance criterion). **Supplemental Figure S2.** Peak activity concentration ratio for the bone-lung phantom as a function of the acquisition time by reference to the 10-min acquisition time PET images separately for the different investigated combinations of voxel sizes, Gaussian filters, and OSEM-TOF (A-D) or OSEM-TOF+PSF (E-H) image reconstructions. Data for all investigated spheres/density regions are presented. Dashed horizontal lines indicate the ±20% deviation (acceptance criterion). **Supplemental Figure S3.** Analysis of correlation and agreement of patient data. Scatter plot (A) and Bland-Altman analysis (B) for SUV_peak_ of reduced acquisition time versus SUV_peak_ of full acquisition time PET images for OSEM-TOF reconstructions. Scatter plot (C) and Bland-Altman analysis (D) for SUV_peak_ of reduced acquisition time versus SUV_peak_ of full acquisition time PET images for OSEM-TOF+PSF image reconstructions.

## Data Availability

The datasets generated and/or analysed during the current study are not publicly available due to privacy legislation but are available from the corresponding author on reasonable request.

## Abbreviations

ACR: Activity concentration ratio
CNR: Contrast-to-noise ratio
EARL: EANM Research Ltd.
FDA: Food and Drug Administration
FWHM: Full width at half maximum
FOV: Field-of-view
HU: Hounsfield units
MIP: Maximum intensity projection
OSEM: Ordered subset expectation maximization
PET/CT: Positron emission tomography / Computed tomography
PET/MR: Positron emission tomography / Magnetic resonance imaging
PSF: Point spread function
PSMA: Prostate specific membrane antigen
PMT: Photomultiplier tube
SiPM: Silicon photomultiplier
SBR: Signal-to-background ratio
SUV: Standardised uptake value
VOI: Volume-of-interest
TOF: Time of flight
