# Supplementary material for: Phantom-based acquisition time and image reconstruction parameter optimisation for oncologic FDG PET/CT examinations using a digital system
Source: BMC Cancer. 2022 Aug 18;22:899. doi: 10.1186/s12885-022-09993-4 (PMC9387080; doi:10.1186/s12885-022-09993-4)
Supplement: Supplementary file 1 — Additional file 1: Supplemental Figure S1. Peak activity concentration ratio for the small-tumour phantom as a function of the acquisition time by reference to the 10-min acquisition time PET images separately for the different investigated combinations of voxel sizes, Gaussian filters, and OSEM-TOF (A-D) or OSEM-TOF+PSF (E-H) image reconstructions. Data for all investigated spheres are presented. Dashed horizontal lines indicate the ±20% deviation (acceptance criterion). Supplemental Figure S2. Peak activity concentration ratio for the bone-lung phantom as a function of the acquisition time by reference to the 10-min acquisition time PET images separately for the different investigated combinations of voxel sizes, Gaussian filters, and OSEM-TOF (A-D) or OSEM-TOF+PSF (E-H) image reconstructions. Data for all investigated spheres/density regions are presented. Dashed horizontal lines indicate the ±20% deviation (acceptance criterion). Supplemental Figure S3. Analysis of correlation and agreement of patient data. Scatter plot (A) and Bland-Altman analysis (B) for SUVpeak of reduced acquisition time versus SUVpeak of full acquisition time PET images for OSEM-TOF reconstructions. Scatter plot (C) and Bland-Altman analysis (D) for SUVpeak of reduced acquisition time versus SUVpeak of full acquisition time PET images for OSEM-TOF+PSF image reconstructions. [file 12885_2022_9993_MOESM1_ESM.pdf]

Supplementary Material to:

Phantom-based acquisition time and image reconstruction parameter optimisation for oncologic FDG PET/CT examinations using a digital system

Pedro Fragoso Costa[1,5], Walter Jentzen[1,5], Alissa Brahmer[1,5], Ilektra-Antonia Mavroeidi[2,5], Fadi Zarrad[1,5], Lale Umutlu[3,5], Wolfgang P. Fendler[1,5], Christoph Rischpler[1,5], Ken Herrmann[1,5], Maurizio Conti[4], Robert Seifert[1,5], Miriam Sraieb[1,5], Manuel Weber[1,5], and David Kersting[1,5]

[1] Department of Nuclear Medicine, University Hospital Essen, West German Cancer Center (WTZ), University of Duisburg-Essen, D-45147 Essen, Germany

[2] Department of Medical Oncology, University Hospital Essen, West German Cancer Center (WTZ), University Duisburg-Essen, D-45147 Essen, Germany

[3] Department of Diagnostic and Interventional Radiology and Neuroradiology, University Hospital Essen, D-45147 Essen, Germany

[4] Siemens Medical Solutions USA, Inc., Knoxville, TN, United States

[5] German Cancer Consortium (DKTK), Partner Site University Hospital Essen, Essen, Germany

Supplemental Figure S1

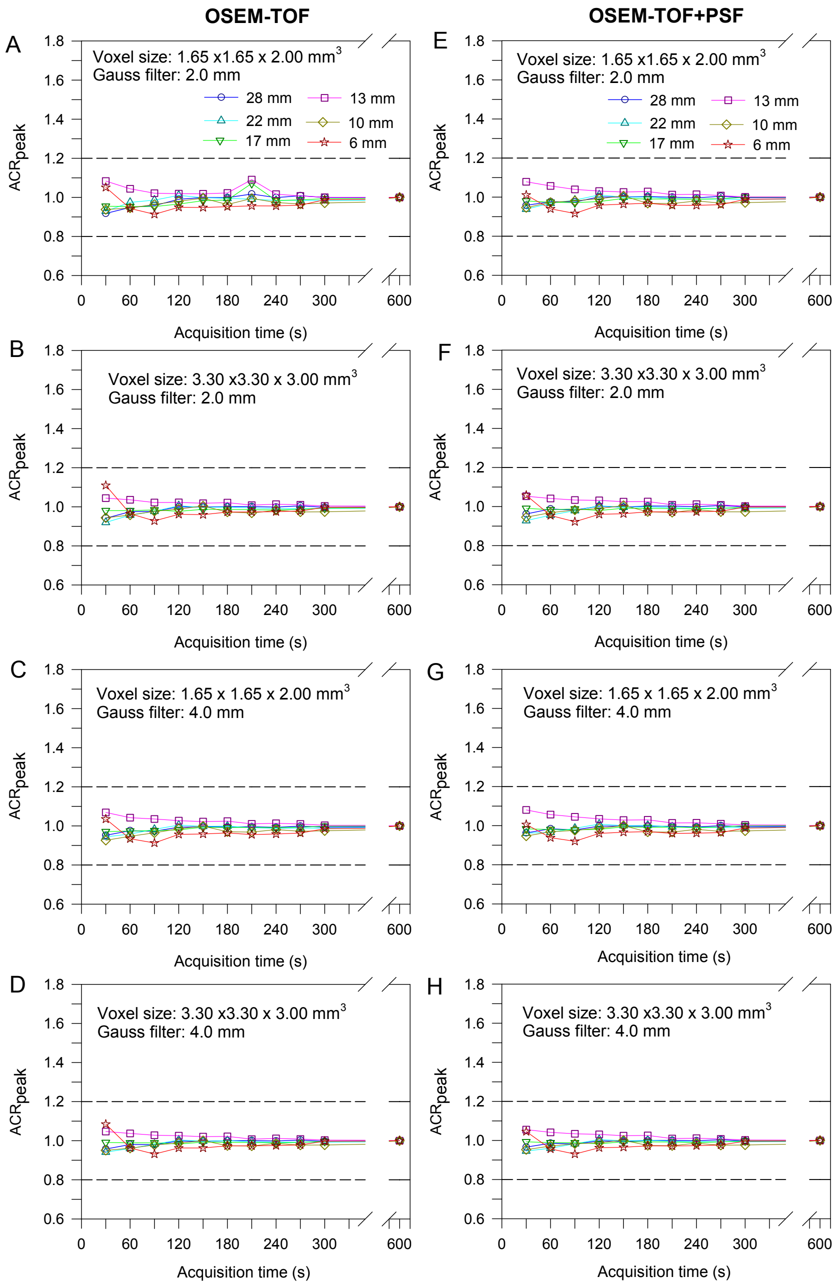

Supplemental Figure S1: Peak activity concentration ratio for the small-tumour phantom as a function of the acquisition time by reference to the 10-min acquisition time PET images separately for the different investigated combinations of voxel sizes, Gaussian filters, and OSEM-TOF (A-D) or OSEM-TOF+PSF (E-H) image reconstructions. Data for all investigated spheres are presented. Dashed horizontal lines indicate the ±20% deviation (acceptance criterion).

Supplemental Figure S2

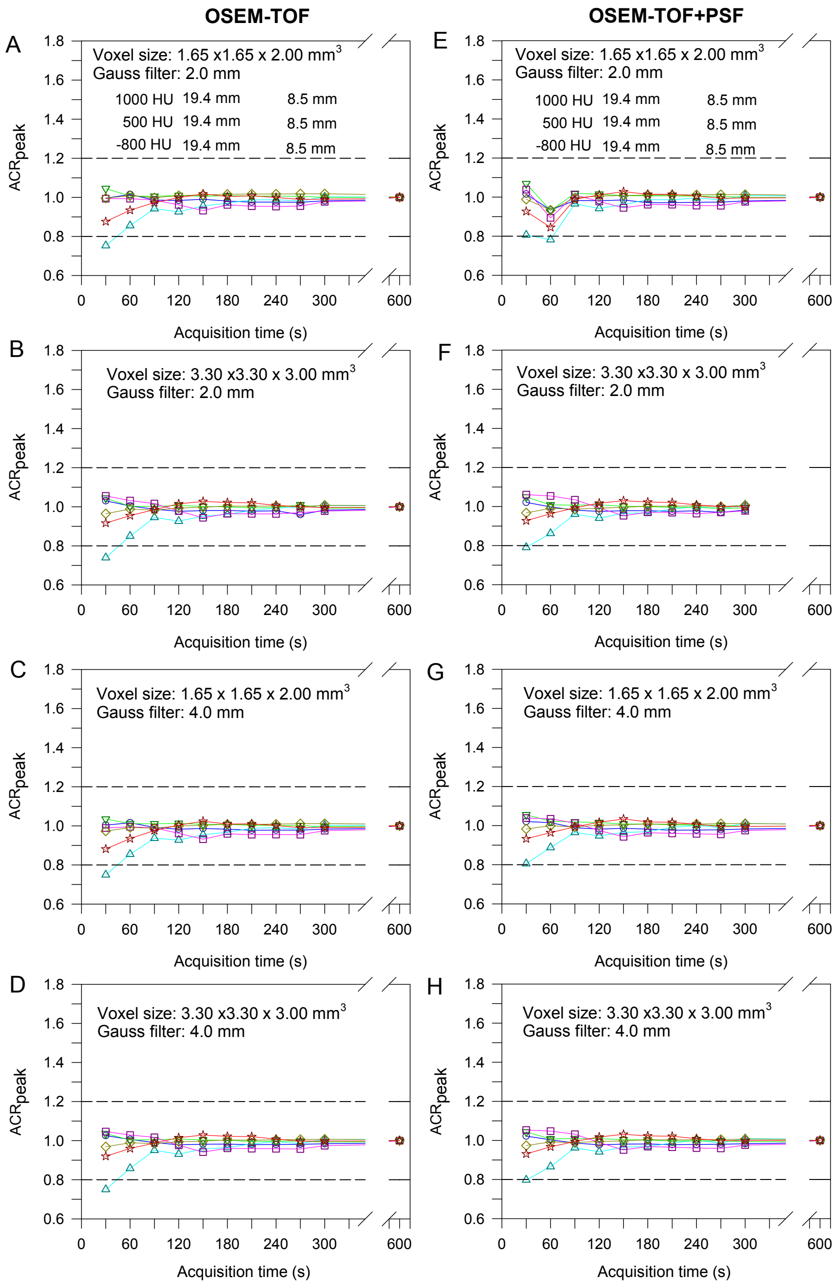

Supplemental Figure S2: Peak activity concentration ratio for the bone-lung phantom as a function of the acquisition time by reference to the 10-min acquisition time PET images separately for the different investigated combinations of voxel sizes, Gaussian filters, and OSEM-TOF (A-D) or OSEM-TOF+PSF (E-H) image reconstructions. Data for all investigated spheres/density regions are presented. Dashed horizontal lines indicate the ±20% deviation (acceptance criterion).

Supplemental Figure S3

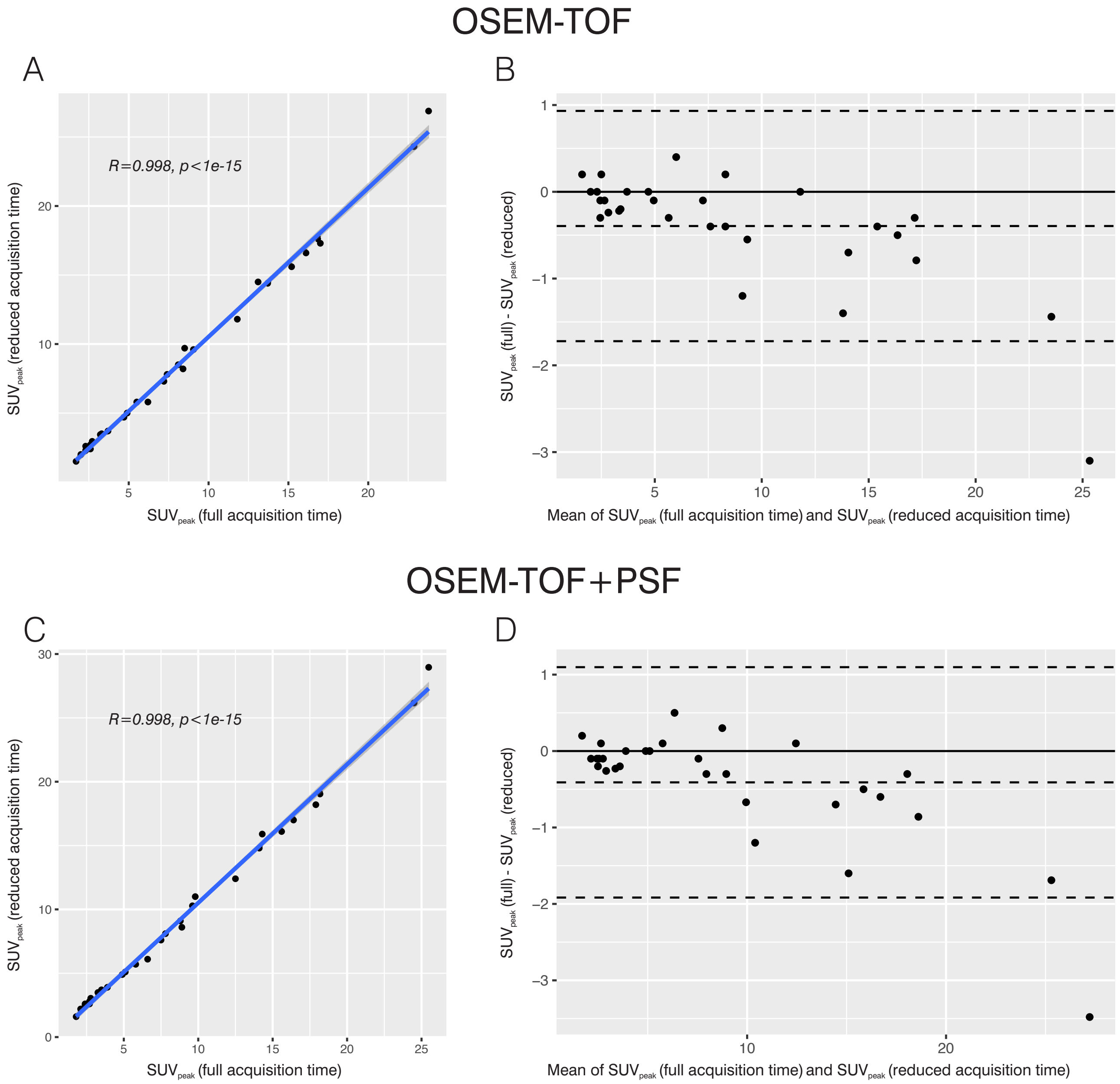

Supplemental Figure S3: Analysis of correlation and agreement of patient data. Scatter plot (A) and Bland-Altman analysis (B) for SUV_{peak} of reduced acquisition time versus SUV_{peak} of full acquisition time PET images for OSEM-TOF reconstructions. Scatter plot (C) and Bland-Altman analysis (D) for SUV_{peak} of reduced acquisition time versus SUV_{peak} of full acquisition time PET images for OSEM-TOF+PSF image reconstructions.
